# Exercise Induces an Augmented Skeletal Muscle Mitochondrial Unfolded Protein Response in a Mouse Model of Obesity Produced by a High-Fat Diet

**DOI:** 10.3390/ijms24065654

**Published:** 2023-03-16

**Authors:** Pía Apablaza, Juan Carlos Bórquez, Rodrigo Mendoza, Mónica Silva, Gladys Tapia, Alejandra Espinosa, Rodrigo Troncoso, Luis A. Videla, Nevenka Juretić, Andrea del Campo

**Affiliations:** 1 Programa de Biología Celular y Molecular, Instituto de Ciencias Biomédicas, Facultad de Medicina, Universidad de Chile, Santiago 8380000, Chile; 2 Laboratorio de Fisiología y Bioenergética Celular, Escuela de Química y Farmacia, Facultad de Química y de Farmacia, Pontificia Universidad Católica de Chile, Santiago 7820436, Chile; 3 Laboratorio de Investigación en Nutrición y Actividad Física (LABINAF), Instituto de Nutrición y Tecnología de los Alimentos (INTA), Universidad de Chile, Santiago 7830490, Chile; 4 Centro de Estudios de Ejercicio, Metabolismo y Cáncer, Programa de Fisiología y Biofísica, Instituto de Ciencias Biomédicas, Facultad de Medicina, Universidad de Chile, Santiago 8380000, Chile; 5 Programa de Farmacología Molecular y Clínica, Instituto de Ciencias Biomédicas, Facultad de Medicina, Universidad de Chile, Santiago 8380000, Chile; 6 Escuela de Medicina, Universidad de Valparaíso, Valparaíso 2340000, Chile

**Keywords:** obesity, UPRmt, skeletal muscle, exercise

## Abstract

Increase in body fat contributes to loss of function and changes in skeletal muscle, accelerating sarcopenia, a phenomenon known as sarco-obesity or sarcopenic obesity. Studies suggest that obesity decreases the skeletal muscle (SM)’s ability to oxidize glucose, increases fatty acid oxidation and reactive oxygen species production, due to mitochondrial dysfunction. Exercise improves mitochondrial dysfunction in obesity; however, it is not known if exercise regulates the mitochondrial unfolded protein response (UPRmt) in the SM. Our study aimed to determine the mito-nuclear UPRmt in response to exercise in a model of obesity, and how this response is associated with the improvement in SM functioning after exercise training. C57BL/6 mice were fed a normal diet and high-fat diet (HFD) for 12 weeks. After 8 weeks, animals were subdivided into sedentary and exercised for the remaining 4 weeks. Grip strength and maximal velocity of mice submitted to HFD improved after training. Our results show an increase in the activation of UPRmt after exercise while in obese mice, proteostasis is basally decreased but shows a more pronounced increase with exercise. These results correlate with improvement in the circulating triglycerides, suggesting mitochondrial proteostasis could be protective and could be related to mitochondrial fuel utilization in SM.

## 1. Introduction

Obesity is considered as one of the 21st century pandemics, and is defined as abnormal or excessive fat accumulation representing a risk to health (WHO). It can be accompanied by the loss of muscle mass which finally leads to a loss of physical capacity, being detrimental to healthspan [1]. At the molecular level, several authors have suggested that a high-fat diet prevents muscle hypertrophy, which causes a loss of muscle strength, decreases the ability to take up and oxidize glucose and increases fatty acid oxidation and reactive oxygen species (ROS) production, due to mitochondrial dysfunction [2,3,4,5]. Within the response mechanisms against mitochondrial damage are (i) mitochondrial biogenesis and mitochondrial dynamics, related to organelle fission, fusion and motility processes to maintain mitochondrial quantity, quality and morphology; (ii) mitophagy, related to the clearance of damaged mitochondria [6,7]; and (iii) the mitochondrial unfolded protein response (UPRmt) related to proteostasis’ maintenance in the mitochondria (mitohormesis). UPRmt is an adaptive response that is conserved from worms to mammals, constituting a first line response to mild mitochondrial stress, which can be due to excessive ROS, proteotoxic stress, depletion of oxidative phosphorylation (OXPHOS) components and depletion of mitochondrial DNA (mtDNA) [8]. Under non-stressful conditions, the Activating Transcription Factor 5 (ATF5) is found in the mitochondria. However, in the presence of stress signals it migrates to the nucleus triggering the transcription of factors associated with mitochondrial proteostasis [9]. Among the transcription factors that have been linked to the UPRmt, ATF5 stands out as the main signaling agent, but also other participants can be listed as UPRmt responders: (i) CEBP Homologous Protein (CHOP), (ii) CCAAT/enhancer binding protein β (CEBPβ), and (iii) Activating Transcription Factor 4 (ATF4) [10,11].

Furthermore, UPRmt involves an increase in various mitochondrial proteases, among which Lon Protease 1 (LONP1) [12] and ClpP/ClpX can be ascribed, as well as characteristic chaperones such as Heat shock protein 90 (Hsp90), mitochondrial Heat shock protein 70 (mtHsp70), Hsp60 and Hsp10 [13,14]. The UPRmt has been described as a beneficial signaling pathway and it has also been linked with improvement in lifespan and in metabolic homeostasis. Nevertheless, a chronic increase in the UPRmt due to the constant presence of a stressor has been suggested to be detrimental to the cell [15,16]. In this regard, skeletal muscle of obese subjects exhibits lower mitochondrial mass, with morphological changes including an increase in mitochondrial fission [17] and impairment of mitochondrial functions. Interestingly, studies in obese humans revealed that exercise reverses these alterations, favoring mitochondrial fusion and improving mitochondrial metabolism, for example, β-oxidation of fatty acids [17,18,19]. On a putative relationship between UPRmt and obesity, it has been suggested that the activation of the UPRmt would be a protective mechanism of obesity or the intake of a high fat diet [20]. For example, mice KO for Surf1, a protein that is part of complex IV of cytochrome C oxidase, show a lower body mass index, fat storage, increased insulin sensitivity and fatty acid oxidation in white adipose tissue [21]. Along with this, Surf−/− mice presented an increased expression of UPRmt-related proteins such as Hsp60, ClpP and Lonp1 in skeletal muscle [22]. In contrast, KO mice for the ClpP gene, which codes for an important UPRmt effector, show a lower prevalence of obesity compared to wild-type ClpP mice and also have improved glucose homeostasis presenting a fasting-like phenotype [23]. According to these considerations, our study assesses the UPRmt response to exercise in a model of high-fat diet and how this response may contribute to the improvement of some aspects of skeletal muscle functioning after training.

## 2. Results

### 2.1. Physical Characteristics Improvement with Exercise after High Fat Diet

**Figure 1:**
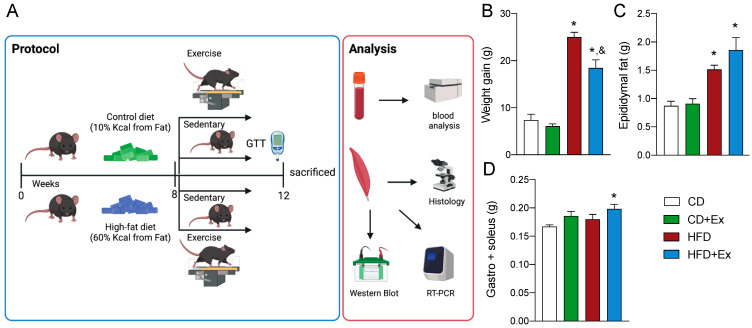
Physical characteristics improve with exercise after HFD. (**A**) Experimental design for C57BL/6 mice, illustration was completed in BioRender. (**B**) Weight gain was significantly increased after HFD. (**C**) Epididymal fat increases with HFD despite exercise training. (**D**) Gastrocnemius/Soleus muscle weight after interventions. Data are presented as mean ± SEM. N = 7 mice per group. Two-way ANOVA followed by Tukey’s post-test. Statistical significance * *p* < 0.05 vs. CD, and ^&^ *p* < 0.05 vs. HFD. CD: Sedentary Control Diet; CD + Ex: Control Diet Exercised; HFD: Sedentary High Fat Diet; HFD + Ex: High Fat Diet Exercised.

Exercise and obesity are two counteractive stressors which produce a mitochondrial response to adapt to the use of the available nutrients. Our model brings together both types of stress, as first the animals were submitted to 8 weeks of high fat diet, with an increase in fat from 10% to 60%, and then a 4-week training program in treadmill exercise (Figure 1A). Weight gain was significantly increased in the HFD group when compared to the control. Noticeably, after 4-week exercise maintaining HFD, weight gain was less than in the HFD group but significantly increased when compared to control mice (Figure 1B). The increase in body weight is mainly due to an increase in adipose tissue. This was observed when epididymal adipose tissue significantly increased in the HFD group independently of exercise training (Figure 1C), while muscle weight (particularly gastrocnemius + soleus) did not change with exercise in Control diet (CD) group but increased in the HFD group (Figure 1D).

### 2.2. Physical Parameters of Mice Submitted to HFD Improve after Exercise Training

**Figure 2:**
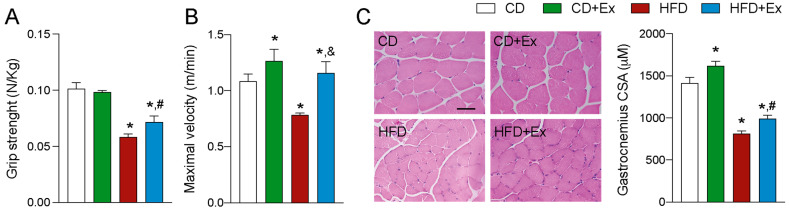
Physical parameters of mice submitted to HFD improve after chronic exercise training. (**A**) Grip Strength was measured in a force gauge and normalized by body weight (N/g). (**B**) Maximal speed was calculated using an incremental speed protocol. (**C**) Histological analysis of the gastrocnemius muscles. Representative image of cross-section gastrocnemius muscle histology stained with Hematoxylin/Eosin, mean of cross-sectional area (CSA) of myofiber quantification (right); 400×. N = 7 mice per group. * *p* < 0.05 vs. CD, ^#^ *p* < 0.05 vs. CD + Ex, and ^&^ *p* < 0.05 vs. HFD. Bar: 20 μm.

Physical performance was also registered. In this regard, grip strength significantly decreased in the HFD group (Figure 2A) with no changes after exercise when compared to CD or HFD. Moreover, there were no changes in strength in the control group after 4-week treadmill exercise. In the treadmill exercise, the maximum speed was significantly decreased in the HFD group but after the training it was recovered to the Control group levels. These results demonstrate that endurance and strength can be handled separately and are differentially affected by both stressors, exercise and HFD (Figure 2B). After physical measurements, histological analysis of the gastrocnemius was performed to correlate the changes in the four groups of animals. Our results show that cross-sectional area of muscle fibers in the gastrocnemius muscle increased after treadmill training when animals were fed with the control diet. The HFD group presented a clear tendency to decrease cross-sectional area, and this was maintained within the group of HFD and exercise training (Figure 2C). This correlates with the results that there was no recovery in force but there was in physical performance.

### 2.3. Biochemical Profile Is Partially Recovered with Exercise after HFD

**Figure 3:**
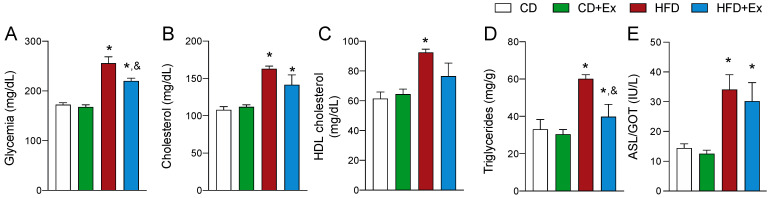
Biochemical profile of HFD and CD mice after chronic exercise. (**A**) Glycemia. (**B**) Total cholesterol. (**C**) HDL-c. (**D**) Triglycerides. (**E**) Plasma levels of GOT. Data are presented as mean ± SEM. N = 7 mice per group. * *p* < 0.05 vs. CD, and ^&^ *p* < 0.05 vs. HFD.

In a biochemical context, lipid profile and fasting glycemia (Figure 3A) were assessed. As expected, the HFD group had an increase in basal glycemia, total cholesterol (Figure 3B), c-HDL levels (Figure 3C) and triglycerides (Figure 3D). After 4 weeks of exercise, the fasting glycemia was reduced in the HFD group. Interestingly, the levels of HDL-c did not change after exercise. Plasma levels of glutamic oxaloacetic transaminase (GOT) were significantly enhanced in the HFD group (Figure 3E) and did not recover after exercise. After the exercise intervention, only the levels of triglycerides recovered in the HFD group (Figure 3D). This could be due to an increase in mitochondrial metabolism and a change in metabolic fuel, as exercise intensity and duration are important determinants of fat oxidation [24].

### 2.4. Differences in the Activation of Mitochondrial Unfolded Protein Response after Diet Distress

**Figure 4:**
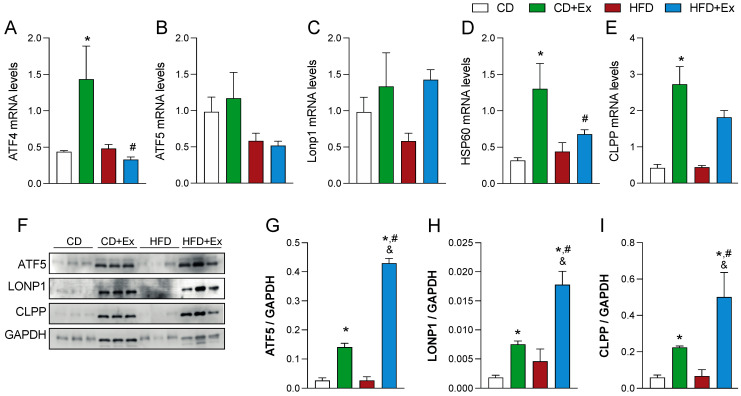
Differences in the activation of Mitochondrial unfolded protein response after diet distress. (**A**–**E**) Real-time PCR for ATF4, ATF5, LONP1, HSP60 and ClpP. PCR amplification of the housekeeping gene GAPDH was performed as a control. (**F**) Representative Western blot for ATF5, LONP1, ClpP and GAPDH. (**G**–**I**) Quantification of protein levels were normalized to GAPDH. N = 7 mice per group * *p* < 0.05 vs. CD, ^#^ *p* < 0.05 vs. CD + Ex, and ^&^ *p* < 0.05 vs. HFD.

As previously mentioned, the UPRmt response is an adaptive mechanism in charge of the maintenance of proteostasis and then contributes to mitochondrial quality control [25,26]. Moreover, mitochondria can adapt to stress situations to modify their metabolism and contribute to cell survival [27]. Our results show that after chronic exercise, both control and HFD conditions did not change mRNA levels of *ATF5* (Figure 4B), while in the HFD group, LONP1 did significantly increase after exercise (Figure 4C). In the same way, mRNA of chaperone *HSP60* and protease *ClpP* increased significantly in control mice after the training (Figure 4D,E), together with an increase in *ATF4* mRNA levels (Figure 4A), while a more discrete increase was found in the HFD group, suggesting there is a difference in mitochondrial adaptations to exercise after being exposed to diet-induced stress. As ATF5 and LONP1 are two principal proteins in the UPRmt signaling pathway, we measured the protein levels to have a complete context of how the UPRmt signaling modifies with stress. Our results show that both LONP1 and ATF5 increase with exercise in both CD and HFD group, being more pronounced in the HFD group (Figure 4F,I). Moreover, ClpP levels also increased after exercise training in the HFD group.

## 3. Discussion

Obesity leads to various changes in the body producing several comorbidities in those who present it. On a molecular level, various studies have shown that different signaling pathways can be modified due to the accumulation of fatty acids, among other signaling molecules that are deregulated with a fat increase in skeletal muscle [28]. Because of the importance of mitochondria in energy metabolism and because of the evidence of mitochondrial damage generated by molecular mechanisms underlying obesity, we assessed the role of mitochondrial response mechanisms upon these stressors. Mito-nuclear imbalance and the UPRmt are two conserved mitochondrial mechanisms that play critical roles in ensuring mitochondrial proteostasis and function. Since UPRmt is a recently described signaling pathway, little is known about its regulation in skeletal muscle in a chronic stress condition such as obesity. Various authors suggest that activation of UPRmt would be a protective factor in a condition of obesity or exposure to a HFD by increasing energy expenditure due to the regulation of metabolism by increasing glycolysis and suppressing oxidative phosphorylation [20]. As reported in studies of surf1 knock-out mice, which have an increased activation of the UPRmt, these mice show lower BMI and fat storage, increased insulin sensitivity and fatty acid oxidation in the adipose tissue [21]. Together with this, an increase in the expression of proteins related to UPRmt such as Hsp60, ClpP and Lonp1 has been observed in both skeletal muscle and heart [22]. In accordance with these results, our findings show a decrease in the mRNA levels of ATF5 in HFD mice when compared to the CD group. In mice fed a low saturated fat diet, it has been described that there is an increase expression of UPRmt related proteins, CliP and HSP60 in adipose tissue, whereas in mice fed a high saturated fat diet there is a decrease in UPRmt and ClpP protein expression, suggesting that activation of ClpP would help to preserve mitochondrial function and efficient fat utilization [20]. On the counterpart, knock-out mice of the ClpP gene, which encodes for an ATP-dependent protease widely distributed in both prokaryotic cells and organelles present in eukaryotic cells, and which is an important UPRmt effector, showed a lower prevalence of the obese phenotype compared with wild-type ClpP mice. In addition, ClpP knock-out mice compared with wild-type mice showed a decrease in insulin resistance, together with improved glucose homeostasis in liver, skeletal muscle and adipose tissue [23,29]. These results suggest there might be a balance in the UPRmt that contributes to metabolic maintenance, where ClpP has an important role. As it has been extensively studied, exercise is one of the best non-pharmacological treatments for obesity, and it also has impact on the skeletal muscle mitochondrial network. In this regard, one of the key adaptations that takes place in skeletal muscle after training is an increase in mitochondrial protein and, therefore, activities of the enzymes of the tricarboxylic acid (TCA) cycle and OXPHOS, changes that can be accompanied by alterations in the mitochondrial network [30,31]. In the context of modifications of mitochondrial proteostasis response, there are a few studies describing high-intensity exercise training [32] and aerobic exercise in the skeletal muscle of aged mice, while another report describes that physical exercise did not change the HSP60 protein content but increased the other two UPRmt markers (LONP1 and YME1L1) in the hypothalamus [33]. Nevertheless, there is no evidence of the activation of these signaling pathways in the skeletal muscle of an obese model. Various studies have described how mitochondrial stress induces proteostasis in the cytosol [34]. A model has recently been proposed linking mitochondrial-mediated regulation of cytosolic translation, folding capacity, ubiquitination, proteasome degradation and autophagy as a multi-layered control of cytosolic proteostasis that overlaps with integrated stress response (ISR) and UPRmt [34]. Because the ubiquitin-proteasome system (UPS) is a central regulator of muscle protein degradation, it has been suggested that alterations in proteolysis would accompany pathological conditions [35]. For example, obese rats present altered UPS, existence of ER stress and upregulation of apoptotic markers in the cerebral cortex, suggesting that UPS could be one of the underlying mechanisms for the neuronal cell death in obese conditions [36]. On the other hand, UPS is required for muscle adaptation following exercise, activating both the proteasome and ubiquitylation to rapidly remove potentially damaged proteins in skeletal muscle [37]. Thus, different quality control mechanisms are integrated and overlap to maintain protein and organellar quality [38]. Our results are then the first to describe the activation of the UPRmt pathway after chronic exercise in a high fat (10% to 60% increase in fat) diet obese mice model of damaged mitochondria. We show that exercise in the skeletal muscle of obese mice induces the UPRmt as observed in an increase of both LONP1 and ClpP protein levels in a more exacerbated way than in CD mice. Altogether, our results indicate that the mitochondrial proteostasis stress in skeletal muscle could be a protective mechanism enhanced with chronic exercise related to mitochondrial fuel utilization in mice. In contrast, in obesity, the mechanism is decreased but still can be activated under exercise stress conditions to improve glucose homeostasis.

## 4. Materials and Methods

### 4.1. Animals

Male mice of the C57BL/6J strain with an initial weight of 12–14 g were used. Mice were maintained in a controlled environment at a constant temperature of 21 °C with free access to water and controlled feeding. The light and dark cycles were of 12 h. Two groups were established with a sample size equal to 7 or 8 per group. Group A corresponded to mice fed a control diet (CD) composed of 10% lipids, 20% proteins and 70% carbohydrates. Group B consisted of mice fed a high fat diet (HFD) composed of 60% lipids, 20% proteins, and 20% carbohydrates (D12492; Research Diets, New Brunswick, NJ, USA). Both groups were fed the respective diet for twelve weeks. All the protocols were approved by the Institutional Animal Care and Use Committee of the Universidad de Chile number 20431-INT-UCH.

### 4.2. Exercise Training Protocol and Treadmill Exhaustion Test

At end of week 8, all mice were acclimated to the treadmill for 10 min during 5 days at a velocity 5–8 m/min with a 5° slope. After, mice performed a graded exercise test to voluntary exhaustion. The test started at 5 m/min for 5 min, followed by speed increment of 1 m/min every 3 min until the animal could no longer keep up with the treadmill speed. The same protocol was performed at the end of the training period. Exercise training on the treadmill was performed for 4 weeks; 5 times per week for 1 h each session at 60–65% of maximal speed reached on treadmill exhaustion test. Finally, mice were sacrificed at least 72 h after the last exercise session.

### 4.3. Strength

Total strength was determined by an all-grip strength test measured in a force gauge (Force Gauge, Dongguan, China). Mice were placed horizontally over the metal grid and pulled by the tail continuously until they could not hold on. This procedure was repeated three times for each mouse to obtain a mean value. Strength determination was performed before and after the training exercise. Grip strength was expressed in Newtons (N) and normalized by body weight (N/g).

### 4.4. RT-qPCR

Total RNA was obtained from skeletal muscle employing Trizol reagent (Ambion, Life Technologies, Waltham, MA, USA) according to the manufacturer’s protocol. The concentration and purity of RNA were determined by absorbance at 260/280 nm. A total of 1 μg of total RNA was reverse transcribed using a high-capacity cDNA reverse transcription kit (Invitrogen, ThermoFisher Scientific, Waltham, MA, USA) according to the manufacturer’s instructions.

Real-time PCR was performed using Stratagene Mx3000P (Stratagene, La Jolla, CA, USA) using Brilliant III Ultra-Fast SYBR QPCR master mix amplification kit (Agilent Technologies, Santa Clara, CA, USA). The primers used were: ATF5 (S) 5′-TGGAGCGGGAGATCCAGTA-3′ (AS) 5′-GACGCTGGAGACAGACAGACGTACA-3′, ATF4 (S) 5′-GGGTTCTGTCTTCCACTCCA-3′ (AS) 5′-AAGCAGCAGAGTCAGGCTTTC-3′; CEBPβ (S) 5′-AACCTGGAGACGCAGCAC-3′ (AS) 5′-ACAGCTGCTCCACCTTCTTC-3′; Hsp60 (S) 5′-CACTGGTATATGGCTCTTGCAC-3′ (AS) 5′-ACTGCTGTCATTGTCCATGC-3′; Lonp1 (S) 5′-CCGGCTGATGTGAATCCTTCT-3′ (AS) 5′-AGCCCTATGTTGGCGTCCTTC-3′ ClpP (S) 5′-CATTCACTGCCCAATTCCAG-3′ (AS) 5′-TGATTTCCTCTGCCTGGATG-3′; GAPDH (S) 5′-TCCGCCCCTTCCGCTGATG-3′ (AS) 5′-CACGGAAGGCCATGCCAGTGA-3′. A typical reaction contained 250 nmol/L of forward and reverse primer, 1 μL cDNA and the final reaction volume was 20 μL. All primers used presented optimal amplification efficiency (between 90% and 110%). PCR amplification of the housekeeping gene GAPDH was performed as a control [39,40]. Thermocycling conditions were as follows: 95 °C for 5 min and 40 cycles of 90 °C for 15 s, 60 °C for 15 s, 72 °C for 15 s. Expression values were normalized to GAPDH and are reported in units of 2^−ΔΔ^CT ± S.D., as described in [33]. CT value was determined by MXPro software when fluorescence was 25% higher than background. PCR products were verified by melting-curve analysis.

### 4.5. Western Blot

Skeletal muscle tissue was lysed with T-PER^TM^ Reagent supplemented with phosphatase and protease inhibitor cocktails. Total protein concentration was determined by BCA assay (Thermo Scientific, USA). Equal amounts of protein were loaded on 10% SDS polyacrylamide gels and then electro-transferred to PVDF membranes. After blocking, primary antibodies for ATF5 (ab184923, ABCAM), LONP1 (ab1411647, ABCAM) and ClpP (ab124822, ABCAM), were incubated overnight at 4 °C, and the appropriate horseradish peroxidase-conjugated secondary antibodies were added. Membranes were incubated with Westar Supernova (Cyanogen, Bologna, Italy), and the luminescence was visualized and digitalized with CDiGit Blot Scanner (Li-Cor Biosciences, Lincoln, NE, USA), and quantified with Image Studio Lite Software (v.5.2; Li-Cor). Protein levels were normalized to GAPDH (ab8245, ABCAM) as reported by [41] in obese models.

### 4.6. Histology

Serial cryosections (12 μm thick) from adult mice muscle were fixed using freshly prepared Paraformaldehyde (4%) in PBS for 30 min and washed in distilled water. Subsequently, Hematoxylin/Eosin techniques were performed to evaluate the general state of muscle tissue. Histological preparations were examined by bright field microscope (Leica dm500) and 10–12 images were obtained of each preparation. These were later analyzed with Image J software (NIH).

### 4.7. Statistical Analysis

Data of *n* mice (*n* = 7–8) were expressed as mean ± SE and analyzed by two-way ANOVA followed by Tukey’s post-test. A *p*-value < 0.05 was considered statistically significant (IC 95%). All statistical analyses were performed using GraphPad Prism 9.

## Data Availability

Not applicable.
